# Assessing the Coverage of Biofortified Foods: Development and Testing of Methods and Indicators in Musanze, Rwanda

**DOI:** 10.1093/cdn/nzaa107

**Published:** 2020-06-18

**Authors:** Nicolai Petry, James P Wirth, Valerie M Friesen, Fabian Rohner, Arcade Nkundineza, Elli Chanzu, Kidist G Tadesse, Jean B Gahutu, Lynnette M Neufeld, Ekin Birol, Erick Boy, Bho Mudyahoto, Tawanda Muzhingi, Mduduzi N N Mbuya

**Affiliations:** GroundWork, Fläsch, Switzerland; GroundWork, Fläsch, Switzerland; Global Alliance for Improved Nutrition (GAIN), Geneva, Switzerland; GroundWork, Fläsch, Switzerland; Sagaci Research, Nairobi, Kenya; Sagaci Research, Nairobi, Kenya; Sagaci Research, Nairobi, Kenya; College of Medicine Health Sciences, University of Rwanda, Kigali, Rwanda; Global Alliance for Improved Nutrition (GAIN), Geneva, Switzerland; HarvestPlus, Washington, DC, USA; HarvestPlus, Washington, DC, USA; HarvestPlus, Washington, DC, USA; International Potato Center, Lima, Peru; Global Alliance for Improved Nutrition (GAIN), Geneva, Switzerland

**Keywords:** biofortification, coverage, Rwanda, iron, beans, orange-fleshed sweet potatoes

## Abstract

**Background:**

Biofortification of staple crops has the potential to increase nutrient intakes and improve health outcomes. Despite program data on the number of farming households reached with and growing biofortified crops, information on the coverage of biofortified foods in the general population is often lacking. Such information is needed to ascertain potential for impact and identify bottlenecks to parts of the impact pathway.

**Objectives:**

We aimed to develop and test methods and indicators for assessing household coverage of biofortified foods.

**Methods:**

To assess biofortification programs, 5 indicators of population-wide household coverage were developed, building on approaches previously used to assess large-scale food fortification programs. These were *1*) consumption of the food; *2*) awareness of the biofortified food; *3*) availability of the biofortified food; *4*) consumption of the biofortified food (ever); and *5*) consumption of the biofortified food (current). To ensure that the indicators are applicable to different settings they were tested in a cross-sectional household-based cluster survey in rural and peri-urban areas in Musanze District, Rwanda where planting materials for iron-biofortified beans (IBs) and orange-fleshed sweet potatoes (OFSPs) were delivered.

**Results:**

Among the 242 households surveyed, consumption of beans and sweet potatoes was 99.2% and 96.3%, respectively. Awareness of IBs or OFSPs was 65.7% and 48.8%, and availability was 23.6% and 10.7%, respectively. Overall, 15.3% and 10.7% of households reported ever consuming IBs and OFSPs, and 10.4% and 2.1% of households were currently consuming these foods, respectively. The major bottlenecks to coverage of biofortified foods were awareness and availability.

**Conclusions:**

These methods and indicators fill a gap in the availability of tools to assess coverage of biofortified foods, and the results of the survey highlight their utility for identifying bottlenecks. Further testing is warranted to confirm the generalizability of the coverage indicators and inform their operationalization when deployed in different settings.

## Introduction

Micronutrient deficiencies are a major public health problem and substantially contribute to the global burden of disease (1, 2). Biofortification, defined as the process of enhancing the micronutrient content in staple foods via selective plant breeding, is a promising approach to help close the micronutrient gap, especially in hard-to-reach populations that are not covered by other nutrition interventions (3, 4). Biofortified foods have been shown to be efficacious in improving the micronutrient status of women and children in controlled trials (5–7) and have potential to increase intake of key micronutrients and reduce the prevalence of micronutrient deficiencies in vulnerable populations (8, 9).

Biofortified crops are steadily being introduced in various countries and the scale of many biofortification programs is increasing (10). HarvestPlus, the leading organization in the development of biofortified crops, has developed tools and methods to assess the number of farming households reached with biofortified crop seeds (11). According to HarvestPlus, ∼8.5 million farming households worldwide were growing and/or consuming biofortified foods in 2019 as a result of their activities (12).

In contrast, data estimating the proportion of nonfarm households consuming biofortified foods are limited. Whereas standardized methods and indicators exist to assess the coverage and consumption of industrially fortified foods at the household level (13, 14), similar methods for biofortified foods have not yet been developed or tested for application in household or market surveys. As the number and scale of programs that deliver biofortified seeds increase, it is critical to monitor the coverage of biofortified foods among both farm and nonfarm households to understand their potential for impact (4, 15), and potential complementarity or overlap with other micronutrient and food system interventions.

The aim of this study was to develop a set of indicators to assess the population coverage of biofortified foods. These indicators were designed to identify bottlenecks to the scale-up of biofortified crops and biofortification programs. In addition, we conducted a household coverage survey to confirm the indicators’ utility. To do so, the household coverage of iron-biofortified beans (IBs) and orange-fleshed sweet potatoes (OFSPs), as examples of biofortified foods *without* visible traits (similar outward appearance to, and barely distinguishable from, the nonbiofortified counterparts) and with visible traits (easily distinguishable from nonbiofortified counterparts due to color change), respectively, was assessed in Musanze district, in the Northern Province of Rwanda.

## Methods

### The program impact pathway for scaling up biofortified foods and corresponding indicators

**FIGURE 1:**
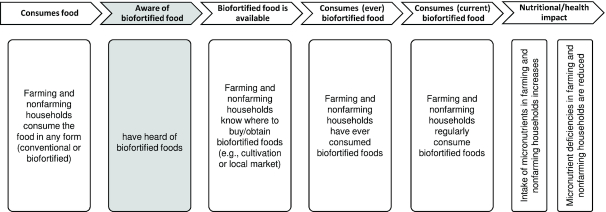
Program impact pathway for biofortified foods.

To develop the coverage indicators, we first articulated the program impact pathway (PIP) underlying the availability of biofortified foods to households, the consumption of the biofortified food, and the achievement of nutritional impacts (**Figure 1**).

**FIGURE 2:**
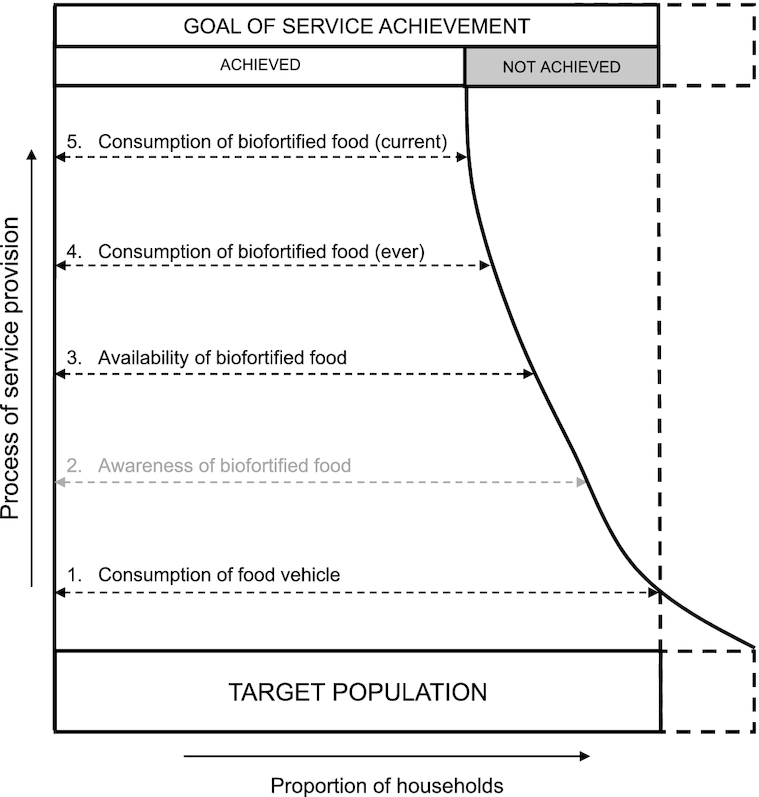
Coverage cascade for biofortified foods. Adapted from Tanahashi (17).

Next, we adapted previously developed indicators for assessing coverage of industrially fortified foods based on the Tanahashi framework of service coverage (16, 17) to the biofortification context. This entailed defining the coverage cascade by identifying the various stages of coverage that should be met before the “goal of service achievement” (i.e., high coverage of the biofortified food) can be reached. Five key stages of coverage were identified, each stage in the cascade depending on the prior stages to be true, with the exception of awareness in some contexts (**Figure 2**).

### Testing of the biofortification coverage indicators

We tested the biofortification coverage indicators in a cross-sectional household survey in Musanze District, Northern Province, Rwanda where 2 programs delivering biofortified planting material had been previously implemented, thereby ensuring the availability of IBs (nonvisible traits) and OFSPs (visible traits).

### Questionnaire development

Formative research activities were conducted in June/July 2019 in Rwanda's Musanze District to collect information needed to inform the design of the questionnaire. This included visits to 10 marketplaces to collect information from all vendors selling beans and/or sweet potatoes (*n* = 114) on availability of the biofortified foods of interest, knowledge about their nutritional value, and insights on customer behavior and purchasing patterns. In addition, 2 focus group discussions (FGDs) were conducted with 20 women (10 in each FGD) to collect information on local terminologies for biofortified foods, availability and consumption of the biofortified foods of interest, as well as preparation practices and storage conditions. A household questionnaire was developed using the Fortification Assessment Coverage Toolkit (FACT) household questionnaire (14) as a template, and modifications to this questionnaire were made based on the information collected through the formative research activities. Questionnaire modules were designed to streamline the interview process and to calculate population-level proportions of the newly developed indicators. The following questions and procedures were used to calculate the 5 key indicators:

1. Consumption of the food: this indicator was based on the response to the question, “Does your household consume [insert food] at home?”
2. Awareness of the biofortified food: this indicator was based on the response to the question, “Have you ever heard of or seen [insert biofortified food]”?
3. Availability of the biofortified food: this indicator was based on the response to the question, “Do you know where to buy/obtain [insert biofortified food]?”
4. Consumption of the biofortified food (ever): this indicator was based on the response to the question, “Have you ever bought/grown/received [insert biofortified food] for eating?”
5. Consumption of the biofortified food (current): for biofortified foods with visible traits (i.e., OFSPs), this indicator was based on a visual confirmation of the biofortified food by the interviewer if it was available in the household. If no food sample was available, classification was based on the response to the question, “The last time your household got [insert biofortified food] for eating, what kind did you get?” after showing the respondent pictures of nonbiofortified and biofortified sweet potatoes. For biofortified foods with nonvisible traits (i.e., IBs), a sample of the food was collected if available in the household and later identified by a breeding specialist. Because few households were able to correctly identify IBs, those households that did not provide a bean sample were excluded from the analyses for this indicator.

Additional questions related to biofortification (e.g., quantity purchased/obtained, purchase frequency, storage and home processing practices, knowledge, attitudes, and practices) and household demographics and socioeconomic status were included in the questionnaire.

### Survey design and participants

A cross-sectional household-based cluster survey was conducted in rural and peri-urban households in August 2019 in Musanze District, Rwanda, where planting material for IBs and OFSPs had been delivered in previous years. The timing of the field work was based on when beans and sweet potatoes were expected to be available based on local planting and harvesting schedules. To calculate the sample size, an intracluster correlation coefficient of 0.1 was assumed, which is based on design effects encountered in FACT surveys. A 2-stage sampling procedure was used. First, 20 rural and 5 peri-urban villages, serving as enumeration areas (EAs), were selected through simple random sampling. In the second stage, households were selected in each EA using probability proportional to size sampling based on the total number of households in the EA. Households in each EA were randomly selected from up-to-date household lists received from the village chiefs. In total, 159 rural and 91 peri-urban households were selected (250 households in total). In each selected household, the person (≥18 y of age) who was most knowledgeable about food purchasing and preparation for the household was invited to participate in the study.

### Data collection

Data were collected by experienced and trained enumerators. The training included classroom instruction, hands-on practice, and 1 d of pilot testing in the field. Data were collected electronically using the Open Data Kit software installed on tablet computers. The questionnaire was programmed in English and Kinyarwanda (local language) to facilitate interview administration. In addition, specific terms and phrases that were difficult to translate from English to Kinyarwanda were discussed in detail during the training to identify consistent language for all interviewers to use for specific questions.

### Data management and statistical analysis

Data were uploaded daily and stored on a password-protected server accessible only to the investigators of the study team. Data consistency checks were done daily to monitor the progress of the field work and check the quality of the data. When data errors were identified, the study team directly interacted with the enumerators to rectify the issues.

Upon completion of data collection, the data were cleaned (e.g., identification and rectification of data entry errors) and any personal identifying information was removed from the database. Before data analysis, we calculated a household wealth index—categorized into quintiles—based on households’ ownership of durable goods, livestock, and the materials used for the floor, walls, and roof of the household's dwelling (18, 19). In addition, indicators of adequacy of each household's sanitation facilities, adequacy of water source, and safety of drinking water were calculated using standardized methods (20). Data analysis was conducted using Stata/IC version 14.2 (Stata Corp.). The statistical precision of prevalences was assessed using 95% confidence limits. The significance of differences between subgroups was tested using the chi-square test.

### Ethics and consent

Ethical approval to conduct the survey was obtained from the National Institute of Statistics of Rwanda (no. 0437/2019/NISR) and the Institutional Review Board of the University of Rwanda, College of Medicine and Health Sciences (no. 367/CMHS IRB/2019). The survey was conducted in accordance with the approved protocol. Written informed consent was obtained from the household interviewees and all parti-cipation was voluntary. If consenting survey participants were illiterate, the consent form was read out loud to them and a witness signature was taken as evidence of consent in lieu of the participant's signature. No compensation was provided for participation in the survey.

## Results

The survey response rate was high (96.8%) with 242 households surveyed, of which 64.5% were rural and 35.5% were peri-urban. Most households had a male household head (72.3%) and, on average, households had 4.3 members. Almost all households had access to a safe water source (94.2%) and, consequently, safe drinking water (96.3%). Conversely, only 42.6% of households had adequate sanitation (i.e., flush or pour flush toilet or pit latrine with slab not shared with another household). Overall, 63.2% of the households owned agricultural land and 43.0% owned livestock, mainly milk cows (35.6%), chicken (27.9%), beef cattle (23.1%), sheep (22.1%), pigs (20.2%), and goats (17.3%).

**Tables 1** and **2** show the 5 measures of coverage for beans and sweet potatoes, respectively. Almost all surveyed households consumed beans and sweet potatoes. Two-thirds of those consuming beans and half of those consuming sweet potatoes were aware of corresponding biofortified varieties. Household respondents reported that they had heard of or seen IBs and OFSPs, most often during village/community meetings or from family, friends, or neighbors. One-quarter of households consuming beans knew where to buy or obtain IBs and just over 10% of households consuming sweet potatoes knew where to buy or obtain OFSPs. Among those households, the market/street stand was the most reported place to obtain IBs and OFSPs followed by from the farmer and, for IBs only, from the shop. Overall, ∼15% of bean-consuming households had ever consumed IBs and almost 10% of households consuming sweet potatoes had ever eaten OFSPs. At the time of the survey ∼10% of households were consuming IBs and ∼2% of households were consuming OFSPs. For both IBs and OFSPs, none of the coverage indicators were associated with rural and peri-urban residence, sex of household head, household land ownership, household wealth quintile, and whether the household grew or purchased the food the last time they got it. However, significantly (*P* < 0.001) more households that grew sweet potatoes in general were aware of (72.2% compared with 42.0%) and knew where to buy/get (22.2% compared with 7.4%) OFSPs than those that did not grow, but bought/obtained nonbiofortified sweet potatoes the last time the household got any sweet potatoes.

**TABLE 1:** Household coverage of IBs in rural and peri-urban households in Musanze, Rwanda^1^

	Total			Peri-urban			Rural			
Indicator	*n*	%^2^	95% CI^3^	*n*	%^2^	95% CI^3^	*n*	%^2^	95% CI^3^	*P* value
Household consumes beans										
Yes	240	99.2	(96.7, 99.8)	84	97.7	(91.1, 99.4)	156	100.0		0.05
No	2	0.8	(0.2, 3.3)	2	2.3	(0.6, 8.9)	0	—		
Household is aware of IBs^4^										
Yes	159	65.7	(59.5, 71.5)	60	69.8	(59.2, 78.6)	99	63.5	(55.6, 70.7)	0.98
No	83	34.3	(28.5, 40.5)	26	30.2	(21.4, 40.8)	57	36.5	(29.3, 44.4)	
Where the respondent heard of IBs^5^										
Village/community meetings	55	34.6	(27.5, 42.4)	14	23.3	(14.3, 35.8)	41	41.4	(32.1, 51.4)	0.002
Relatives/friends/neighbors	45	28.3	(21.8, 35.9)	20	33.3	(22.5, 46.2)	25	25.3	(17.6, 34.8)	0.28
Health extension workers	10	6.3	(3.4, 11.4)	3	5.0	(1.6, 14.5)	7	7.1	(3.4, 14.2)	0.60
Community leaders	13	8.2	(4.8, 13.6)	2	3.3	(0.8, 12.5)	11	11.1	(6.2, 19.1)	0.09
Marketplace/shop	11	6.9	(3.8, 12.1)	6	10.0	(4.5, 20.7)	5	5.1	(2.1, 11.7)	0.24
IBs are available to the household^6^										
Yes	57	23.6	(18.6, 29.4)	25	29.1	(20.4, 39.6)	32	20.5	(14.9, 27.6)	0.14
No	185	76.4	(70.6, 81.4)	61	70.9	(60.4, 79.6)	124	79.5	(72.4, 85.1)	
Where the household can buy/get IBs^7^										
Shop	14	24.6	(14.9, 37.7)	5	20.0	(8.3, 40.7)	9	28.1	(15.0, 46.4)	0.49
At the farmgate	17	29.8	(19.1, 43.3)	6	24.0	(10.9, 44.9)	11	34.4	(19.8, 52.6)	0.40
Market/street stand	43	75.4	(62.3, 85.1)	18	72.0	(51.1, 86.4)	25	78.1	(60.1, 89.4)	0.60
Moving street vendor	2	3.5	(0.8, 13.5)	1	4.0	(0.5, 24.7)	1	3.1	(0.4, 20.1)	0.86
Household ever consumed IBs^8^										
Yes	37	15.3	(11.3, 20.4)	14	16.3	(9.8, 25.7)	23	14.7	(10.0, 21.3)	0.75
No	205	84.7	(79.6, 88.7)	72	83.7	(74.3, 90.2)	133	85.3	(78.7, 90.0)	
Household currently consumes IBs^9^										
Yes	21	10.4	(6.9, 15.5)	7	10.9	(5.3, 21.3)	14	10.1	(6.1, 16.5)	0.86
No	181	89.6	(84.5, 93.1)	57	89.1	(78.7, 94.7)	124	89.9	(83.5, 93.9)	

1 The *n*s are unweighted denominators for each subgroup; subgroups that do not sum to the total have missing data. IB, iron-biofortified bean.

2 Percentages are unweighted to account for equal probability of selection.

3 CIs calculated taking into account the simple random sampling design.

4 Households that reported not being aware of IBs and households that reported not consuming beans were classified as “No.”

5 Includes only households that were aware of IBs; respondents were able to provide multiple responses.

6 Households that did not know where to buy/obtain IBs and households that reported not consuming beans were classified as “No.”

7 Includes only households that knew where to buy/obtain IBs; respondents were able to provide multiple responses.

8 Households that did not consume beans and households that never consumed IBs were classified as “No.”

9 Households that did not consume beans and households that did not consume IBs at the time of the survey were classified as “No.”

**TABLE 2:** Household coverage of OFSPs in rural and peri-urban households in Musanze, Rwanda^1^

	Total			Peri-urban			Rural			
Indicator	*n*	%^2^	95% CI^3^	*n*	%^2^	95% CI^3^	*n*	%^2^	95% CI^3^	*P* value
Household consumes sweet potatoes										
Yes	233	96.3	(93.0, 98.1)	81	94.2	(86.7, 97.6)	152	97.4	(93.3, 99.0)	0.20
No	9	3.7	(1.9, 7.0)	5	5.8	(2.4, 13.3)	4	2.6	(1.0, 6.7)	
Household is aware of OFSPs^4^										
Yes	118	48.8	(42.5, 55.1)	44	51.2	(40.6, 61.6)	74	47.4	(39.7, 55.3)	0.58
No	124	51.2	(44.9, 57.5)	42	48.8	(38.4, 59.4)	82	52.6	(44.7, 60.3)	
Where the respondent heard of OFSPs^5^										
Village/community meetings	34	28.8	(21.3, 37.7)	14	31.8	(19.7, 47.0)	20	27.0	(18.0, 38.4)	0.58
Relatives/friends/neighbors	27	22.9	(16.1, 31.5)	10	22.7	(12.6, 37.6)	17	23.0	(14.7, 34.1)	0.98
Health extension workers	23	19.5	(13.2, 27.8)	10	22.7	(12.6, 37.6)	13	17.6	(10.4, 28.1)	0.47
Community leaders	9	7.6	(4.0, 14.1)	2	4.5	(1.1, 16.7)	7	9.5	(4.5, 18.7)	0.34
Women groups	6	5.1	(2.3, 11.0)	0	—		6	8.1	(3.6, 17.1)	0.05
Marketplace/shop	21	17.8	(11.8, 25.9)	7	15.9	(7.7, 30.0)	14	18.9	(11.4, 29.6)	0.68
Agricultural extension staff	7	5.9	(2.8, 12.0)	2	4.5	(1.1, 16.7)	5	6.8	(2.8, 15.4)	0.63
Radio	12	10.2	(5.8, 17.2)	5	11.4	(4.7, 24.8)	7	9.5	(4.5, 18.7)	0.74
OFSPs are available to household^6^										
Yes	26	10.7	(7.4, 15.3)	9	10.5	(5.5, 19.0)	17	10.9	(6.9, 16.9)	0.92
No	216	89.3	(84.7, 92.6)	77	89.5	(81.0, 94.5)	139	89.1	(83.1, 93.1)	
Where the household can buy/obtain OFSPs^7^										
Shop	0	0.0	—	0	0.0	—	0	0.0	—	—
At the farmgate	11	42.3	(24.2, 62.8)	4	44.4	(16.4, 76.6)	7	41.2	(19.9, 66.3)	0.88
Market/street stand	19	73.1	(51.7, 87.3)	7	77.8	(39.4, 95.0)	12	70.6	(44.0, 88.0)	0.70
Moving street vendor	0	0.0	—	0	0.0	—	0	0.0	—	—
Household ever consumed OFSPs^8^										
Yes	25	10.3	(7.1, 14.9)	8	9.3	(4.7, 17.6)	17	10.9	(6.9, 16.9)	0.70
No	217	89.7	(85.1, 92.9)	78	90.7	(82.4, 95.3)	139	89.1	(83.1, 93.1)	
Household currently consumes OFSPs^9^										
Yes	5	2.1	(0.9, 4.9)	1	1.2	(0.2, 7.9)	4	2.6	(1.0, 6.7)	0.47
No	237	97.9	(95.1, 99.1)	85	98.8	(92.1, 99.8)	152	97.4	(93.3, 99.0)	

1 The *n*s are unweighted denominators for each subgroup. OFSP, orange-fleshed sweet potato.

2 Percentages are unweighted to account for equal probability of selection.

3 CIs calculated taking into account the simple random sampling design.

4 Households that reported not being aware of OFSPs and households that reported not consuming sweet potatoes were classified as “No.”

5 Includes only households that were aware of OFSPs; respondents were able to provide multiple responses.

6 Households that did not know where to buy/obtain OFSPs and households that reported not consuming sweet potatoes were classified as “No.”

7 Includes only households that knew where to buy/obtain OFSPs; respondents were able to provide multiple responses.

8 Households that did not consume sweet potatoes and households that never consumed OFSPs were classified as “No.”

9 Households that did not consume sweet potatoes and households that did not consume OFSPs at the time of the survey were classified as “No.”

**FIGURE 3:**
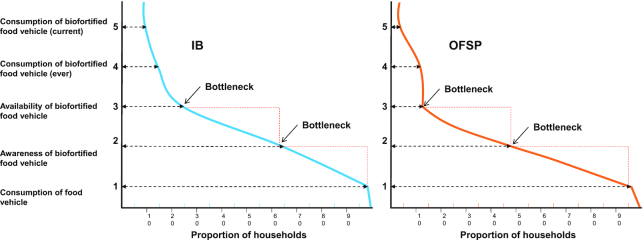
Bottlenecks in coverage of IBs (A) and OFSPs (B) among households in Musanze, Rwanda. IB, iron-biofortified bean; OFSP, orange-fleshed sweet potato.

Using the new model for assessing the coverage of biofortified foods, the main bottlenecks identified were awareness and availability of the biofortified food vehicle for both IBs and OFSPs (**Figure 3**). The largest difference in the proportion of households achieving each stage of coverage drops between the household consuming the food (in any form) and being aware of the biofortified food: i.e., a drop in coverage of about one-third for IBs and ∼50% for OFSPs. The next largest decline in coverage is between awareness and availability of the biofortified foods: i.e., a drop in coverage of ∼40% for both IBs and OFSPs.

**Table 3** shows bean identification by the household respondent and the breeding specialist. A large proportion of the respondents were not able to identify IBs correctly. Of the 10.2% of households actually consuming IBs (as identified by the breeding specialist), only ∼25% correctly reported that the type of beans they consumed were biofortified, whereas ∼65% reported consuming nonbiofortified beans and the remainder did not know what kind of beans they consumed.

**TABLE 3:** Household bean identification

		Bean type identified by breeding specialist	
		Biofortified beans	Nonbiofortified beans
Bean type presumed by household respondent	Biofortified beans	2.5%	5.4%
	Nonbiofortified beans	6.4%	78.8%
	Don't know	1.3%	5.6%
	Total	10.2%	89.8%

## Discussion

We developed a theory-based coverage cascade for biofortified foods and 5 corresponding indicators for assessing coverage. This framework is designed to identify bottlenecks to consumption of biofortified foods across different stages of the PIP. Because HarvestPlus programs focus primarily on delivering seeds/planting material to farmers, the performance of their programs is assessed by measuring the seed coverage of farmers only. Although this coverage approach is suitable to estimate the number of farming households that grow biofortified foods, it is not sufficient for estimating the proportion of *all* households in a certain area that consume biofortified foods. As such, the PIP developed here represents a more comprehensive tool for measuring program performance. Because the indicators are theory-based, they can be used to measure the performance of all biofortified foods with visible or nonvisible traits. Our experience in Rwanda confirms the utility of these indicators for assessing coverage of, and for identifying bottlenecks to scaling up, biofortified foods with visible traits (i.e., OFSPs) and nonvisible traits (i.e., IBs).

The 5 measures of coverage presented here for biofortified foods are based on the Tanahashi coverage framework and build on the model for assessing industrially fortified foods (16, 17). However, some important differences exist between large-scale food fortification (LSFF) and biofortification that required further adaptation. In both LSFF and biofortification, the first indicator in the Tanahashi model of coverage is the same: the consumption of the food in any form [(bio-)fortified or conventional]. Apart from this indicator, the LSFF Tanahashi model (21) and the biofortification Tanahanshi model vary considerably. In LSFF, the second coverage indicator is the consumption of the *fortifiable* food (or food product that is industrially produced). LSFF programs are essentially implemented at the level of industrial food producers, such as large flour mills and vegetable oil refineries, thus, the maximum proportion of households that have potential to benefit from the program are those consuming industrially fortified food. This indicator is not applicable in the context of biofortification because biofortification occurs at the seed level and thus the proportion of households that have potential to benefit from the program are those consuming the food in any form. In LSFF, the third coverage indicator is consumption of the fortified food, which is determined objectively by brand identification and/or laboratory analysis to confirm the presence of the added nutrient in food samples collected from households or markets. Oftentimes laboratory analyses are quantitative and measure both the presence of a nutrient and its concentration, and therefore also assess the consumption of a food that is fortified to standard (i.e., adequately) as a fourth LSFF coverage indicator.

This fourth LSFF indicator is analogous to the biofortification framework's fifth coverage indicator (i.e., current consumption of the biofortified food). Assessment of the current household consumption of biofortified foods can be determined objectively through visual assessment by the interviewer at the household for biofortified foods with visible traits, e.g., OFSPs. For biofortified foods with nonvisible traits that remain nearly identical to the nonbiofortified version, visual confirmation is possible in some cases by breeding experts (e.g., beans) or by farmers familiar with particular plant and/or pod characteristics, but may not be feasible for other crops (e.g., rice, wheat, pearl millet, white zinc maize). In these cases, quantitative laboratory testing may be needed for confirmation, which could provide additional information on the concentration of micronutrients.

To address this important gap in the coverage cascade for food biofortification between consumption of the food vehicle and consumption of the biofortified food vehicle, we included 3 additional indicators aimed at understanding the drivers of consumption and to provide additional information for identifying bottlenecks to scale-up. These are awareness, availability, and consumption of the biofortified food (ever). Fundamentally, for households to switch from the nonbiofortified food or product made from the food to the biofortified variety they are required, in most circumstances, to be aware of the biofortified alternative and for it to be available either for home growing or purchase through the market. However, availability in the community or within the household does not necessarily translate to consumption among individuals (22) because after contact with the biofortified food (ever consumed), a positive experience with the crop (e.g., yield in case of farming households), and/or food acceptance (e.g., sensory attributes, cooking behavior, nutritional or health benefits) are critical for consumers to consume the food regularly. It is also important to note that certain linkages within the PIP for biofortified foods will be more or less important in different food systems and dietary pattern contexts. For example, awareness may or may not be a prerequisite for delivery models depending on the maturity of the program (e.g., early adoption compared with mainstreamed) and whether the biofortified food has nonvisible or visible traits of being biofortified. With nonvisible traits, the biofortified food is otherwise indistinguishable from the conventional varieties, making awareness a less important part of the PIP. Alternatively, if the biofortified food has visible and perhaps unfamiliar traits (e.g., changes in color, taste, or texture), additional information will have to be provided to consumers via strategies such as behavior change communication (BCC) to communicate the benefits of the biofortified varieties. As such, awareness can be a part of the PIP or an effect modifier translating availability to uptake. It should be noted that these additional indicators can also be used in LSFF programs, but are less important given the ability to easily assess consumption of the fortifiable and fortified foods and use that information to identify critical bottlenecks in the program delivery.

In Musanze, almost all households were found to consume beans and sweet potatoes, which validates the premise of biofortification, i.e., targeting key staples that are consumed regularly by all households (3). However, the IB and OFSP coverage (i.e., current consumption of the biofortified foods) was relatively low; and awareness and availability of IBs and OFSPs were identified as the main bottlenecks. Despite the large drop from consumption of the foods in any form (indicator 1) to awareness of the biofortified food (indicator 2), awareness figures are considerable for IBs and OFSPs because seeds were introduced as recently as 2012. As already mentioned above, in biofortification the utility of the awareness indicator varies largely depending on the nature of the food vehicle. For beans, awareness might not necessarily be a prerequisite for promoting consumption because a large proportion of households did not know that they were consuming biofortified beans. On the other hand, the higher price of IBs was identified as 1 of the reasons more bean vendors did not sell biofortified varieties, rendering awareness creation a necessity to increase consumers’ willingness to pay more and with it drive the demand for IBs. A study conducted in Rwanda indicated that people might be willing to pay more for certain biofortified bean varieties (23); however, strategies to lower the price of biofortified varieties, in addition to awareness creation, also need to be explored. That said, if biofortified varieties with nonvisible traits were to replace all conventional varieties available in the market, coverage would be easier to scale up and monitor and awareness creation or education on the benefits may not be needed. For OFSPs, which are visibly different than other sweet potato varieties, awareness has been shown to be a driver of consumption/adoption aside from other factors such as organoleptic characteristics, taste preferences, and access to planting material (24). To further increase awareness, multiple and strategic awareness campaigns and other information-sharing efforts targeting consumers and farmers could be implemented, which would likely have a beneficial effect on the adoption rate and demand creation (3, 25, 26). Those campaigns could entail BCC activities to promote the production and consumption of biofortified crops as well as educational activities and messages related to positive health behaviors and child care and feeding practices (10).

The main bottleneck to coverage of both biofortified foods was their availability; only about every fifth and every tenth household reported knowing where to buy/obtain IBs or OFSPs, respectively. Biofortification of staple foods can be regarded as a sustainable approach if the delivery model also involves developing new markets by adding value through the development of new products (27). However, sustained consumption is influenced most directly by ensuring supply of planting material and demand for the biofortified food (22). Thus, it should be evaluated if seed multiplication and delivery systems are still functioning such that consumer demand can be satisfied. Further, availability of crops (biofortified and conventional) can be substantially influenced by seasonality and local planting and harvesting schedules, thus, the timing of the field work of future surveys should be taken into account during the surveys’ design phase.

A limitation of the survey was that not all households had beans at home at the time of the survey and only 9 out of 10 households provided a bean sample. Because the identification of IBs proved to be difficult for most of the respondents, only those households providing a bean sample were included in the analysis. Therefore, the indicator on consumption of the biofortified food (currently) may be slightly under- or overestimated. Future surveys should consider arranging for field workers to make repeated visits to households in order to get a sample from all households.

The survey results show that the developed tool fills a gap in the availability of methods to assess household coverage of biofortified foods. A major strength of the tool is its utility to assess the coverage of biofortified foods and to identify bottlenecks to the “scale-up” of biofortification programs. Although the developed tool has been designed to be applicable in any setting and for any biofortified foods with visible or nonvisible traits, further testing is warranted to confirm the generalizability of the indicators when applied to different contexts.

## Notes

This study was supported by Bill & Melinda Gates Foundation (BMFG), and some funds from BMGF contributed to the salaries of staffers from GroundWork, Sagaci Research, and University of Rwanda personnel.

Author disclosures: the authors report no conflicts of interest.

Abbreviations used: BCC, behavior change communication; EA, enumeration area; FACT, Fortification Assessment Coverage Toolkit; FGD, focus group discussion; IB, iron-biofortified bean; LSFF, large-scale food fortification; OFSP, orange-fleshed sweet potato; PIP, program impact pathway.
